# The association between serum uric acid and glaucoma severity in primary angle closure glaucoma: a retrospective case-control study

**DOI:** 10.18632/oncotarget.13745

**Published:** 2016-12-01

**Authors:** Shengjie Li, Mingxi Shao, Binghua Tang, Aiping Zhang, Wenjun Cao, Xinghuai Sun

**Affiliations:** ^1^ Department of Clinical Laboratory, Eye and ENT Hospital, Shanghai Medical College, Fudan University, Shangai, China; ^2^ Department of Ophthalmology and Visual Science, Eye and ENT Hospital, Shanghai Medical College, Fudan University, Shangai, China; ^3^ State Key Laboratory of Medical Neurobiology, Institutes of Brain Science and Collaborative Innovation Center for Brain Science, Fudan University, Shanghai, China; ^4^ Key Laboratory of Myopia, Ministry of Health (Fudan University), Shangai, China; ^5^ Shanghai Key Laboratory of Visual Impairment and Restoration (Fudan University), Shangai, China

**Keywords:** primary angle closure glaucoma, serum, uric acid, oxidative stress

## Abstract

Uric acid (UA) is a major antioxidant molecule and has been hypothesized to have a protective effect on the central nervous system against oxidative damage. We prospectively investigated the serum concentration of UA in primary angle closure glaucoma (PACG), and explored the association between serum concentration of UA and the severity of PACG. Using a retrospective case-control study design, 886 PACG subjects and 994 control subjects who attended the Eye & ENT Hospital of Fudan University, were eligible for this study. Glaucoma severity was classified as mild (MD ≤ 6.00 dB), moderate (12 dB ≥ MD > 6 dB) and severe (MD > 12 dB) based on the MD (mean deviation). The levels of UA were significantly lower (*p* = 0.025) in PACG (0.286 ± 0.082 mmol/l) compared with control (0.295 ± 0.085 mmol/l). The mean serum UA levels were lowest in the severe group (0.281 ± 0.074 mmol/l) followed by moderate (0.282 ± 0.080 mmol/l) and mild (0.297 ± 0.090 mmol/l) with significant differences among the three groups (*p* = 0.032). In multivariate regression analysis, there was a significant negative correlation between UA level and vertical cup-disc ratio (B = −0.165, *p* = 0.035). Significantly lower serum UA concentration in PACG and its negative association with disease severity presented it as an important candidate in reaction to oxidative stress in glaucoma pathogenesis.

## INTRODUCTION

Primary glaucoma is a progressive optic neuropathy and one of the leading causes of global irreversible blindness, and the number of people (aged 40–80 years) with glaucoma worldwide is projected to increase from 76.0 million in 2020 and 111.8 million in 2040 [1–3]. Although chamber angle closure is known to be one of the key risk factors for PACG, oxidative stress and several other indispensible factors may also contribute [4–14].

As the final product of the common pathway of purine metabolism, UA is a major antioxidant with metal-chelating properties [15] as well as the ability to scavenge nitrogen radicals and superoxide which help block the generation of strong oxidant peroxynitrite [16]. UA has been hypothesized to have a protective effect on the central nervous system against oxidative damage [17, 18]. Lolekha P et al. [19] and Vieru E et al. [20] reported that patients with Parkinson's disease showed significantly lower serum UA and uric acid/creatinine (UA/Cr) ratio than control. Whether serum UA levels are changed and involved in the pathophysiological mechanisms of PACG remained unclear. Babizhayev MA et al. [21] reported that prevention of oxidative stress exposure to the trabecular meshwork with a N-acetylcarnosine ophthalmic prodrug of carnosine and oral formulation of non-hydrolizedcarnosine may help to reduce the progression of glaucoma. Moreover, several studies suggested that markers of oxidative stress including chemia-modified albumin, protein nitrotyrosine, lipid oxidation products and 8-hydroxydeoxyguanosin, increased significantly in glaucoma [7, 22–25]. However, whether peripheral blood UA concentration and UA/Cr ratio were changed in PACG and associated with the pathogenesis of glaucoma still remained to be studied.

The objective of this study was to measure serum UA and creatinine concentration and to further explore the relationships between UA and UA/Cr ratio and glaucoma severity in PACG.

## RESULTS

### Characteristics of the study patients

A total of 886 PACG subjects and 994 control subjects from the Eye & ENT Hospital of Fudan University were eligible for the study from January 2010 to December 2015. Only one eye was selected randomly if both eyes suffered from PACG. There was no statistical difference in the mean age and gender between the PACG and control group (*p* > 0.05). The mean serum levels of UA and UA/Cr ratio were significantly lower in the PACG compared with the control group (*p* < 0.001). PACG group had a higher serum creatinine concentration compared with the control group (*p* < 0.001). The demographic, Cr, UA, UA/Cr ratio of the PACG and control groups were summarized in Table 1.

**Table 1:** Demographics, creatinine, UA, and UA/Cr ratio of subjects with PACG

Factors	PACG group	Control group	*t* value	*P* value
Age (year)	63.17 ± 10.65	63.26 ± 10.12	0.199	0.842
Gender (male/female)	302/584	370/624	2.008	0.156
Creatinine (umol/l)				
Total	69.54 ± 17.61	67.34 ± 20.45	2.478	0.013
Male	80.41 ± 16.51	77.84 ± 19.85	3.009	0.003
Female	63.92 ± 15.39	61.12 ± 18.14	2.879	0.004
UA (mmol/l)				
Total	0.286 ± 0.082	0.295 ± 0.085	2.236	0.025
Male	0.327 ± 0.078	0.342 ± 0.088	2.274	0.023
Female	0.260 ± 0.068	0.268 ± 0.069	1.972	0.049
UA/Cr ratio				
Total	4.23 ± 1.20	4.51 ± 1.24	3.709	< 0.001
Male	4.17 ± 1.15	4.53 ± 1.31	3.716	< 0.001
Female	4.25 ± 1.23	4.51 ± 1.19	3.703	< 0.001

Abbreviations: UA: uric acid, Data are expressed as mean ± standard deviation (SD).

Independent-Samples *T* Test and chi-square test were used.

### Comparison of UA, creatinine, UA/Cr ratio and ocular parameters in subjects with PACG, stratified according to severity

**Figure 1:**
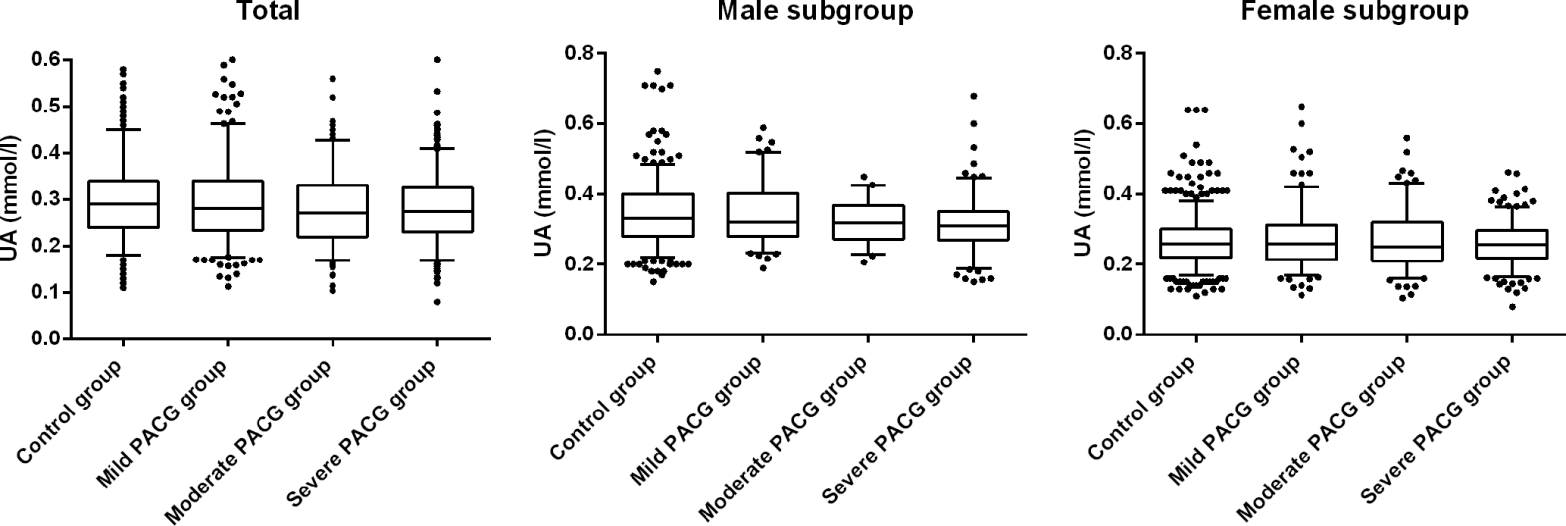
Comparison of serum levels of uric acid (UA) in patients with mild, moderate, severe PACG and control group: using box-and-whisker plot The box contained 50% of all values (from 25th to 75th percentile) and was divided by the horizontal bar of the median value (50th percentile). The whiskers showed the remainder of the distribution (1.5 × Inter Quartile Range). Outliers were shown as dots.

Based on the MD, the PACG subjects were categorized into 3 subgroups of different severity level of which 286 were classified as mild, 198 as moderate and 402 as severe. There was no statistical difference in the mean age and gender (*p* = 0.605, *p* = 0.063, respectively) among the three groups. The mean serum levels of UA was lowest in the severe PACG group, followed by moderate PACG and mild PACG, and the differences among groups were significant (*p* = 0.032). The mean serum level of creatinine was not significantly different among the three groups (*p* > 0.05). A similar trend was observed when UA were compared among the 3 groups with respect to gender. The ocular parameters were significantly different among the three groups (*p* < 0.05). The level of UA, creatinine, UA/Cr ratio, and ocular characteristics of the three groups were shown in Table 2 and Figure 1.

**Table 2:** Comparison of demographics, creatinine, UA, UA/Cr ratio and ocular parameters in subjects with PACG, stratified according to severity

	Mild PACG, *n*= 286	Moderate PACG, *n* = 198	Severe PACG, *n*= 402	*P* value
Age, years	62.79 ± 10.57	63.77 ± 10.87	63.14 ± 10.61	0.605
Female, N (%)	186 (65.03)	144 (73.73)	254 (63.18)	0.063
SBP (mm Hg)	131.40 ± 14.82	130.20 ± 13.37	131.01 ± 14.60	0.671
DBP (mm Hg)	76.39 ± 8.39	76.54 ± 7.95	76.18 ± 9.62	0.891
BMI, Kg/m^2^	23.19 ± 3.30	22.45 ± 2.92	22.79 ± 4.08	0.285
IOP (mm Hg)	28.80 ± 10.55	30.49 ± 11.46	36.78 ± 13.11	< 0.001^a,c^
VCDR	0.46 ± 0.17	0.59 ± 0.20	0.81 ± 0.20	< 0.001^a,b,c^
CCT (mm)	547.52 ± 55.21	537.66 ± 43.11	543.91 ± 50.66	0.201
ACD (mm)	1.80 ± 0.46	1.85 ± 0.44	1.85 ± 0.45	0.341
AL (mm)	22.16 ± 1.17	22.29 ± 0.94	22.55 ± 1.43	0.001^a,c^
MD (dB)	3.85 ± 1.83	9.20 ± 2.15	21.65 ± 4.71	< 0.001^a,b,c^
MS (dB)	20.52 ± 7.77	17.83 ± 2.79	5.56 ± 4.77	< 0.001^a,b,c^
Creatinine (umol/l)				
Total	71.53 ± 20.13	68.70 ± 16.18	68.53 ± 16.22	0.065^a^
Male	85.22 ± 21.10	82.92 ± 13.64	76.29 ± 12.51	< 0.001^a,b^
Female	64.18 ± 15.18	63.50 ± 13.75	63.96 ± 16.44	0.921
UA (mmol/l)				
Total	0.297 ± 0.090	0.282 ± 0.080	0.281 ± 0.074	0.032^a,b^
Male	0.342 ± 0.085	0.322 ± 0.058	0.317 ± 0.079	0.040^a^
Female	0.273 ± 0.083	0.267 ± 0.083	0.258 ± 0.061	0.049^a^
UA/Cr ratio				
Total	4.25 ± 1.21	4.23 ± 1.29	4.22 ± 1.14	0.948
Male	4.15 ± 1.14	3.97 ± 0.89	4.24 ± 1.23	0.332
Female	4.31 ± 1.25	4.33 ± 1.40	4.18 ± 1.09	0.384

Abbreviations: UA: uric acid, SBP: systolic blood pressure, DBP: diastolic blood pressure, BMI: body mass index, IOP: intraocular pressure, VCDR: vertical cup-disc ratio, CCT: central corneal thickness, AL: axial length, ACD: anterior chamber depth, MD: visual fields mean deviation, MS: visual fields mean sensitivity, PACG: primary angle closure glaucoma. Data are expressed as mean ± standard deviation (SD). Chi-square test and One-way ANOVA was used.

^a^*P* < 0.05 for the difference between Mild PACG and Severe PACG (1-way ANOVA with the LSD post hoc test).

^b^*P* < 0.05 for the difference between Mild PACG and Moderate PACG (1-way ANOVA with the LSD post hoc test).

^c^*P* < 0.05 for the difference between Moderate PACG and Severe PACG (1-way ANOVA with the LSD post hoc test).

### Spearman correlation between UA and ocular parameters

Table 3 demonstrated the Spearman correlations of UA with the ocular parameters. In the overall PACG group, a statistically significant negative correlation between UA and VCDR (vertical cup-disc ratio) (*r* = −0.111, *p* = 0.001), and positive correlation between UA and AL (axial length) (*r* = 0.151, *p* < 0.001) were observed. In the mild PACG group, a negative correlation between UA and VCDR (*r* = −0.308, *p* < 0.001), UA and MD (*r* = −0.242, *p* = 0.013), and positive correlation between UA and AL (*r* = 0.142, *p* = 0.025), UA and MS (mean sensitivity) (*r* = 0.471, *p* < 0.001) were observed. In the moderate PACG group, the correlations between UA and AL, UA and MD, UA and MS were also statistically significant. A similar trend was observed when correlation among UA and ocular parameters were performed with respect to gender.

**Table 3:** Spearman correlation between UA and ocular parameters

	Spearman Correlation (*p* value)				Male PACG Spearman Correlation (*p* value)		Female PACG Spearman Correlation (*p* value)		
	Overall	Mild	Moderate	Severe	Mild	Severe	Overall	Mild	Moderate
VCDR	−0.111 (0.001)	−0.308 (< 0.001)	NS	−0.136 (0.009)	−0.371 (< 0.001)	−0.214 (0.011)	−0.169 (< 0.001)	−0.211 (0.005)	NS
AL	0.151 (< 0.001)	0.142 (0.025)	0.233 (0.002)	NS	NS	NS	NS	NS	0.182 (0.039)
MD	NS	−0.242 (0.013)	−0.169 (0.047)	NS	−0.308 (0.050)	NS	NS	NS	NS
MS	NS	0.471 (< 0.001)	0.209 (0.014)	NS	−0.549 (< 0.001)	NS	NS	0.305(0.016)	0.226 (0.020)

Abbreviations: UA: uric acid, VCDR: vertical cup-disc ratio, AL: axial length, MD: visual fields mean deviation, MS: visual fields mean sensitivity, NS: not significant. PACG: primary angle closure glaucoma. Male PACG Spearman correlation: no statistically significant correction between UA and ocular parameters in overall and moderate subgroup. Female PACG Spearman correlation: no statistically significant correction between UA and ocular parameters in severe subgroup.

### Multiple linear regressions for associations between UA and ocular parameters

Table 4 demonstrated the multiple linear regressions of UA with the ocular parameters. In multivariate regression analysis after adjusting for age, gender, blood pressure, and BMI, there was a statistically significant negative correlation between UA and VCDR (B = −0.165, *p* = 0.035) in the overall PACG patients. In the mild PACG group, a negative correlation between UA and VCDR (*r* = −0.156, *p* = 0.026), and positive correlation between UA and MS (*r* = 0.341, *p* = 0.046) were observed. In the severe PACG group, a positive correlation between UA and VCDR (*r* = −0.150, *p* = 0.044) was observed. A similar trend was observed when association among UA and ocular parameters were performed by multiple linear regressions with respect to gender.

**Table 4:** Multiple linear regressions for associations between UA and ocular parameters

PACG B (*p* value)					Male PACG B (*p* value)		Female PACG B (*p* value)		
	Overall	Mild	Moderate	Severe	Mild	Severe	Overall	Mild	Moderate
VCDR	−0.165 (0.035)	−0.156 (0.026)	NS	−0.150 (0.044)	NS	−0.284 (0.020)	−0.209 (0.040)	−0.420 (0.001)	NS
AL	NS	NS	0.259 (0.025)	NS	NS	NS	NS	NS	NS
MS	NS	0.341(0.046)	NS	NS	−0.502 (0.020)	NS	NS	NS	0.239 (0.049)

Abbreviations: UA: uric acid, VCDR: vertical cup-disc ratio, AL: axial length, MS: visual fields mean sensitivity, NS: not significant. PACG: primary angle closure glaucoma. Analysis adjusted for age, gender, blood pressure, and BMI. Male PACG: no statistically significant correction between UA and ocular parameters in overall and moderate subgroup. Female PACG: no statistically significant correction between UA and ocular parameters in severe subgroup.

## DISCUSSION

To the best of our knowledge, this study has been the first to focus on the investigation of serum UA and UA/Cr ratios in PACG and further evaluate their relationship with disease severity. The principal finding was that serum UA levels and UA/Cr ratios in PACG patients were lower compared with sex and age-matched control group subjects, in addition, UA levels were found to be associated with the severity of PACG. The serum UA/Cr ratio was used to reduce the possible interference caused by sex and differences in renal function [20]. These data suggested a possible role of UA in the pathogenesis of PACG.

The levels of peripheral blood UA have been reported to be associated with hypertension [26] and Alzheimer's disease [27]. Oxidative stress played a role in neuron cell death and contributes to the pathogenesis of these diseases [17]. Oxidative stress was also considered to contribute to the physiologic alterations in aqueous humor outflow and subsequently to IOP elevation and retinal ganglion cell (RGC) damage in glaucoma [13, 28].

In this study, we found significantly lower serum UA levels in PACG patients compared with control group in both male and female subjects. However, Yuki K et al. [29] reported that higher serum uric acid levels was found in their normal tension glaucoma subjects in comparison to their control subjects. We speculate that the difference in disease etiology may explain the different findings. Erdurmus M et al. [30] and Ferreira SM et al. [31] reported that serum level of total antioxidant capacity was decreased in patients with primary open-angle glaucoma and pseudoexfoliation glaucoma. In addition, aqueous humor UA levels were significantly lower in the congenital glaucoma rabbit model compared with the age-matched controls [32]. Sorkhabi R et al. [22] reported a significant correlation between a higher aqueous humor 8-hydroxydeoxyguanosin level and lower antioxidant capacity levels in the serum of patients with glaucoma. Therefore, the serum antioxidant capacity could reflect the local ocular redox status [33]. We suggest that the decreased level of serum UA may play a part in the anti-oxidative insufficiency which could contribute to PACG development.

There have been reports suggesting that glaucomatous damage is associated with episodes of systemic inflammation which are likely to cause a systemic decrease in UA [34]. Oxidative stress, inflammatory response, infection and cell death appear to be correlated. Oxidative stress induced an inflammatory response that interfered with the homeostasis of the cell [35]. Several inflammatory molecules such as IL-6, IL-8, IL-1alpha, ELAM-1 were up-regulated which may also be induced by oxidative stress [35]. Astafurov K et al. [34] reported that patients with glaucoma had higher bacterial oral counts compared to control subjects and lipopolysaccharide administration in glaucoma animal models resulted in enhancement of axonal degeneration and neuronal loss. In addition, some studies reported that infection of H. pylori had a statistically significant association with glaucoma [36, 37]. Being one of the main anti-oxidants, UA might be consumed by oxidizing agents to prevent an inflammatory response, as mentioned by the above studies. Therefore, we suspect that systemic inflammation and oxidative stress were likely to cause an obvious consumption of UA and significant decrease of its serum level.

Moreover, the decreased serum levels of UA in PACG patients were an outcome of an oxidative stress-related process in the optic nerve system, eye tissue and possibly in other tissues which meant that this decrease was likely to be a result of the direct reaction between UA and oxidizing agents [27, 38]. Sacca SC et al. [10] reviewed that oxidative free radicals and reactive oxygen species were able to affect the cellularity of the human trabecular meshwork. The above studies indicated that glaucoma patients were under a high oxidation state both in the eye and body. Therefore, it is possible that UA would be consumed in the process of PACG by preferentially reacting with oxidizing agents both in the eye and peripheral blood.

*In vitro* studies showed that oxidative stress accelerated cell apoptosis and led to accumulation of extra cellular matrix in the trabecular meshwork, which could both increase the resistance of the aqueous humor outflow and result in IOP elevation and later visual field loss [23, 39, 40]. Our previous work showed that chronic exposure of angular aqueousplexus cells to oxidative stress decreased cell monolayer permeability and up-regulated cytoskeletal and cell–cell adhesion protein expression [14]. Hooper DC et al. [41] reported that mouse exhibited diminished signs (piloerection, tail paralysis, hind limb paralysis, moribund) and better survival in a multiple sclerosis disease model which was treated with UA injection. Scott GS et al. [42] reported that UA played an important role in the protection against peroxynitrite-mediated pathogenesis of inflammatory CNS disorders. In cellular models of neuronal death induced by glutamate exposure or oxygen–glucose deprivation, the exogenous administration of UA reduced neuronal damage, suppressing the accumulation of ROS [43]. Therefore, UA has been hypothesized to have a protective effect on the central nervous system against oxidative damage [17, 44]. These results led us to speculate that lower levels of UA may be risk factors of glaucomatousoptic neuropathy.

Moreover, based on the MD, the PACG subjects were categorized into 3 subgroups of different severity level (mild, moderate, and severe). The mean serum levels of UA were lowest in the severe PACG group, followed by moderate PACG and mild PACG. However, the mean serum level of UA was not statistically different between moderate and severe PACG group (Figure 1 and Table 2). This suggests that UA might play an important role in the beginning of the disease process, and the role of the UA decreases in more advanced stages of the disease. There were also statistically significant negative correlations between UA and VCDR, UA and MD, and a positive correlation between UA and MS. Since the correlation may also be affected by other factors, multivariate analysis was conducted to further investigate the association between UA and glaucoma. Statistical data showed that there was also a significant correlation between UA and VCDR, and between UA and MS. We speculated that the oxidative stress system was increasingly activated along with the progression of PACG while the serum level of UA decreased accordingly. In conclusion, we hypothesize that the mean serum level of UA was associated with PACG severity. This predictive association between serum UA level and PACG severity further indicated the potential role of UA as an important anti-oxidative content in the disease pathogenesis. This finding was novel and potentially very important. The exploration for the role of UA and oxidative stress in PACG appeared to be quite difficult. The mechanism of UA in the pathogenesis of glaucomatous optic neuropathy was worth further exploration in our follow-up study.

Moreover, Table 1 highlighted significant difference in UA between PACG and control groups. We found that the difference between men in PACG and control groups in UA levels was only 0.015. However, the difference between females and males was much higher: in the PACG group it was 0.067 (4-fold higher); in the control group it was 0.074 (5-fold higher). Recently, population-based surveys of glaucoma in Asians reported that the prevalence of PACG was higher in the female than male population [45, 46]. Cheng JW et al. [46] found that PACG affects women 1.51 times more frequently than men. Therefore, our hypothesis was that the much lower UA level might be the cause for the female to have a much higher risk of PACG.

Although this is the first study which focused on the evaluation of serum level of UA, UA/Cr ratios and its relationship between PACG and disease severity, we acknowledged that our present study has some limitations. (1) We did not consider treatment with brinzolamide eye drops, carbonic anhydrase inhibitor, and mannitol and how it might affect renal function. In this study, there was no statistical difference of creatinine among the mild, moderate, and severe PACG subgroups. The serum UA/Cr ratio was analyzed considering the existence of possible interference such as medication and difference in renal function. UA/Cr ratios in PACG patients were also lower compared with the control group. (2) Owing to this study was a retrospective case-control study, information on ocular parameters in controls subjects was lacking.

The present study demonstrated that the mean serum level of UA was decreased and negatively associated with PACG severity which suggested the possible association between UA with the development of glaucoma and the involvement of oxidative stress in the pathogenesis of PACG.

## MATERIALS AND METHODS

### Patients

This was a retrospective case-control study design. The study was approved by the Ethics Committee of the Eye-ENT Hospital of Fudan University, Shanghai, China and was conducted according to the Declaration of Helsinki. Written informed consent for the use of any clinical data in research was obtained for all patients at the time of admission to the Eye-ENT Hospital of Fudan University. PACG subjects and control subjects who attended the Eye-ENT Hospital of Fudan University from January 2010 to December 2015 were selected according to the inclusion criteria listed below.

### Recruitment procedures

The data of all the PACG subjects (*n* = 1350) during January 2010 through December 2015 and control subjects (*n* = 1645) were collected from the inpatient electronic database. 446 PACG subjects and 595 control subjects were excluded according to the inclusion criteria. The exclusion rate of PACG and control group was 33.04% and 36.17% respectively. A further 56 control subjects were randomly excluded because they were not age and sex matched to the PACG group. At last, a total of 886 PACG subjects and 994 control subjects from the Eye & ENT Hospital of Fudan University were eligible for the study.

### Inclusion criteria

### PACG subjects

(1) PACG subjects were selected from inpatients. Each subject underwent a standardized ophthalmic examination, which included refractive status, slit-lamp biomicroscopy, fundus examination, IOP, CCT (central corneal thickness), AL, ACD (anterior chamber depth), visual field examination, and gonioscopy, performed by glaucoma specialists. MD (visual field–derived mean deviation) and MS (mean sensitivity) were measured by Octopus automated perimetry (HAAG, STREIT, Switzerland). IOP was measured using Goldmanapplanation tonometry. Fundus photography was performed with a retinal camera (TRC-NW200, Topcon). A-scan ultrasound (A-Scan Pachymeter, Ultrasonic, Exton, PA, USA) was used to measure AL, ACD, and CCT.

(2) PACG was diagnosed on the basis of narrow angles with glaucomatous optic neuropathy with corresponding visual field loss. This was defined as glaucomahemifield test outside normal limits including a cluster of three or more, non-edge, contiguous points on the pattern deviation plot, not crossing the horizontal meridian with a probability of less than 5% of being present in the age-matched normal (one of which was less than1%), an abnormal pattern standard deviation with P less than 5% occurring in the normal population, and fulfilling the test reliability criteria (fixation losses less than 20%, false positives less than 33% and/or false negatives less than 33%). PACG was diagnosed in eyes with narrow angles, with elevated IOP (IOP >21 mm Hg); at least 180 of angle-closure obliterating the pigmented part of the trabecular meshwork, whether synechial or appositional, segmented or continuous; and eyes in which the degree of peripheralanteriorsynechial is too extensive to be managed by laser peripheral iridotomy. [4, 47, 48] Newly diagnosed PACG patients and referral PACG patients were included. Subjects receiving glaucoma medications were also included. Patients who were taking medications which influence serum uric acid level were excluded. Patients were divided into three groups with different severity based on MD, i.e. mild (MD ≤ 6.00 dB), moderate (12 dB ≥ MD >6 dB) and severe (MD > 12 dB) [49, 50].

(3) PACG subjects had no other ocular diseases and systemic diseases such as hyperuricemia, diabetes, cardiac, autoimmune disease and cancer.

### Control subjects

Control subjects were composed of patients with strabismus (*n* = 168), amblyopia (*n* = 146), nystagmus (*n* = 111), epicophosis (*n* = 144), vocal cords leukoplakia (*n* = 163), lacrimal ducts obstruction (*n* = 155), and nasal septal deviation (*n* = 107). They were selected from the inpatients record at the Eye-ENT Hospital of Fudan University from January 2010 to December 2015. Each subject underwent a standardized physical examination and preliminary ophthalmic examinations, which included refractive status, slit-lamp, and IOP, performed by glaucoma specialists. IOP was measured using Goldmanapplanation tonometry. All the control subjects were excluded from any family history of glaucoma and systemic diseases which included but not restricted to hyperuricemia, diabetes, cardiac, autoimmune disease and cancer.

### UA and creatinine

In this study, PACG and control subjects were selected from the hospital inpatients. As part of a standard care at the Fudan's University Eye & ENT Hospital, peripheral blood samples were routinely collected from all the inpatients and tested for UA levels. Quantification of serum UA and creatinine was measured by enzymatic colorimetry using a commercially available kit (Roche Diagnostics GmbH, Mannheim, Germany). Internal controls were analyzed daily over the 6-year period, with typical monthly CVs of 2%–4% and no significant changes in the values. The reference ranges for UA between males and females were different [51, 52]. The reference range of UA was 0.2023–0.4165 mmol/l for males and 0.1428–0.3392 mmol/l for females. Therefore, the subjects were categorized into gender subgroups.

### Data analysis

The data was analyzed by SPSS13.0 (SPSS Inc., Chicago, IL). UA and creatinine concentrations were presented as mean ± standard deviation (SD). UA/Cr ratio = (UA*1000)/Cr. Normality was assessed with the Kolmogorov-Smirnoff test. The independent student's *t*-test and chi-square test were used for comparison of characteristics of patients between the PACG group and control group. The one-way ANOVA test was used to compare the levels of serum UA, serum creatinine, UA/Cr, and ocular parameters among the three groups with different severity. The associations between UA and ocular parameters in PACG were analyzed by Spearman correlation. After Spearman correlation, multivariate linear regression analysis (adjusting for age, gender, blood pressure, and BMI (body mass index) to prevent bias) was performed to evaluate the association between UA and disease severity, namely visual field indices (MD, MS), VCDR, and other ocular parameters. A *P* value of less than 0.05 was considered statistically significant.
